# Bactericidal Effects against *S. aureus* and Physicochemical Properties of Plasma Activated Water stored at different temperatures

**DOI:** 10.1038/srep28505

**Published:** 2016-06-27

**Authors:** Jin Shen, Ying Tian, Yinglong Li, Ruonan Ma, Qian Zhang, Jue Zhang, Jing Fang

**Affiliations:** 1 Institute for Environmental Health and Related Product Safety, Chinese Center for Disease Control and Prevention, Beijing 100000, China; 2 Academy for Advanced Interdisciplinary Studies, Peking University, Beijing 100000, China; 3 Academy for Advanced Interdisciplinary Studies, Peking University, Beijing, College of Engineering, Peking University, Beijing 100000, China

## Abstract

Water activated by non-thermal plasma creates an acidified solution containing reactive oxygen and nitrogen species, known as plasma-activated water (PAW). The objective of this study was to investigate the effects of different storage temperatures (25 °C, 4 °C, −20 °C, −80 °C) on bactericidal activities against *S. aureus* and physicochemical properties of PAW up to 30 days. Interestingly, PAW stored at −80 °C yielded the best antibacterial activity against *Staphylococcus aureus*, 3~4 log reduction over a 30-day period after PAW generation; meanwhile, PAW stored at 25 °C, 4 °C, and −20 °C, respectively, yielded 0.2~2 log decrease in cell viability after the same exposure and storage time. These results were verified by scanning electron microscope (SEM). The physicochemical properties of PAW stored at different temperatures were evaluated, including pH, oxidation reduction potential (ORP), and hydrogen peroxide, nitrate, nitrite anion and NO radical levels. These findings suggested that bacterial activity of PAW stored at 25 °C, 4 °C, −20 °C decreased over time, and depended on three germicidal factors, specifically ORP, H_2_O_2_, and NO_3_^−^. Moreover, PAW stored at −80 °C retained bactericidal activity, with NO_2_^−^ contributing to bactericidal ability in association with H_2_O_2_. Our findings provide a basis for PAW storage and practical applications in disinfection and food preservation.

Plasma-activated water (PAW), prepared by non-thermal plasma treatment of distilled water, has gained increasing attention as an aqueous disinfectant. Various plasma sources are used to activate water, including direct current (DC), low-frequency discharge, radio frequency discharge, pulsed coronas, dielectric barrier discharge, atmospheric pressure plasma jets, and microwave discharge^1^,^2^,^3^,^4^. PAW can efficiently inhibit a wide range of microorganisms such as *Hafnia alvei*, *Escherichia coli*, *Saccharomyces cerevisiae*, *Staphylococcus aureus*, and *Candida albicans*^5^,^6^,^7^,^8^,^9^. The main advantages of using PAW for bacterial inhibition include less adverse impact on the environment, and no need for transportation and storage of potentially hazardous chemicals; indeed, chemical sanitizers, especially chlorine-based products, have raised increasing public health concerns about the risk of carcinogenic chlorinated organic compounds^10^. As a green disinfection product, PAW is a promising alternative to traditional sanitizers applied in agriculture (sterilization of fruits and vegetables) and food industry (disinfection of poultry products). R. Ma^11^ reported the potential of PAW for inhibiting *S. aureus* inoculated to strawberries. In addition, Y. Xu^12^ pointed out that PAW soaking is a promising method for fresh-keeping of postharvest *A. bisporus*.

Based on discharge type, working gas, and the chemical composition of the surrounding environment, various chemical reactions induced by plasma can be initiated, with a number of resulting primary and secondary species penetrating or dissolving into the liquid. Among the chemical species in PAW, OH· radical, atomic oxygen, ozone, and hydrogen peroxide are considered the main reactive oxygen species (ROS), playing a dominant role in the inactivation process^13^,^14^. The contribution of nitrogen-based reactive species also has to be considered: nitric oxide and its derivatives formed with water, including nitrites, nitrates and peroxynitrites^15^,^16^,^17^,^18^,^19^. Synergistic effects of reactive species are possible in plasma-treated liquid for microbial inhibition. Indeed, the antimicrobial properties of PAW were tentatively attributed to the synergetic effects of acidic pH and nitrite/nitrate remaining in noticeable concentrations in the solution^6^,^8^,^19^. Reactive-nitrogen- and -oxygen-based species play an important role in the toxic effect of PAW, and have DNA, RNA, proteins, and lipids as principal targets^20^,^21^. Moreover, both non-thermal plasma and PAW could catalyze the generation of intracellular ROS, consequently causing oxidative stress in bacterial cells^22^,^23^,^24^.

However, active species in PAW are difficult to assess due to short life spans and fast disproportionation in the plasma-liquid systems. Studies evaluating the effect of atomic oxygen on bacterial killing in water are scarce, since excited atomic oxygen only has a life time of ~30 ns^25^. It is also worth noting that trace amounts of ozone (a few ppm) were detected in air^26^. In water, ozone can live for 1 000 s at room temperature^27^. OH, with extreme reactivity, can be easily scavenged by virtually any organic molecule in its close vicinity, which accounts for its short life-span of about 10^−9^s^28^. Exploring PAW stability, Matthew J Traylor^8^ pointed out the complexity of PAW solutions, where multiple chemical components exert varying biological effects in differing time scales, after measuring physicochemical properties and bacterial inhibition over a 7-day period.

Storage conditions are important factors affecting the physicochemical properties and bactericidal activity of PAW. However, little information is available concerning the effects of storage conditions such as temperature, lighting, and sealing. The aim of this study was to investigate the physicochemical properties and bactericidal activities of PAW stored at different temperatures. The effects of storage temperatures on pH, ORP, hydrogen peroxide, nitrate and nitrite anion levels and the stability of bactericidal efficiency of PAW were assessed for up to 30 days after generation. PAW’s antimicrobial efficacy against *S. aureus* was examined by colony forming unit (CFU) count, and further verified by SEM. OES was employed to detect the main excited reactive species in PAW. Moreover, PAW’s physicochemical characteristics, including pH, ORP, and hydrogen peroxide, nitrate and nitrite anion levels, were recorded. Finally, ESR spectroscopy was used to detect NO radicals in PAW. Understanding the physicochemical properties and bactericidal activity of PAW before and after storage is important to the practical application in inactivating harmful microorganisms in food industries and agriculture.

## Experimental Section

### Plasma Microjet Device and PAW Generation

**Figure 1:**
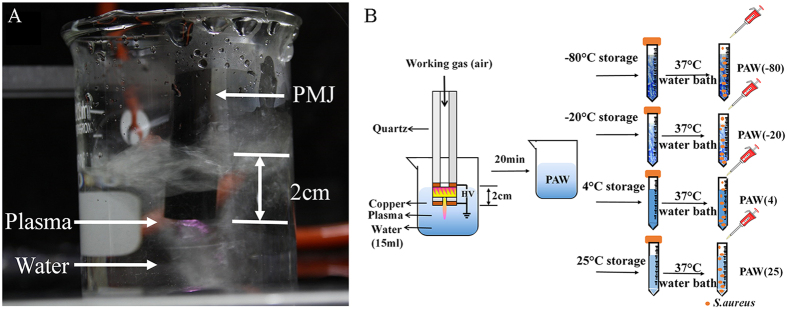
(**A**) Image of PAW generation, and (**B**) schematic diagram of plasma jet and experimental arrangement, including PAW generation and storage at different temperatures.

The air plasma generator is schematically illustrated in Fig. 1(A), and was designed based on the HEDBS structure^29^. The system, mainly consisting of copper electrodes and quartz dielectric, was set at the end of a quartz tube (inlet diameter, 1.5 mm). Air with 260 L/h gas flow rate was injected into the quartz tube, and the high voltage electrode connected to a power source (20 kHz). A homogeneous plasma was generated in the discharge gap of 0.5 mm and a plasma jet reaching 7 mm long ejected through the end outlet of 0.5 mm. All device parts were fixed to each other to prevent accidental displacement.

As shown in Fig. 1(B), PAW was produced by placing the plasma jet beneath the water surface. The distance between the PMJ end and liquid surface was 20 mm. Every 10 ml sterile distilled water was activated by plasma for 20 min to obtain PAW, which was stored in centrifuge tubes. Four different storage temperatures were adopted, including 25 °C, 4 °C, −20 °C, and −80 °C for 1, 3, 7, 15, and 30 days, respectively. For simplified description of the different PAW states, PAW(25), PAW(4), PAW(−20), and PAW(−80) represented PAW stored at 25 °C, 4 °C, −20 °C, and −80 °C after plasma activation, respectively. Meanwhile, PAW-0 referred to water without plasma activation.

### Bacterial strains, growth conditions, and PAW treatment

*S. aureus* (China General Microbiological Culture Collection Center, CGMCC number 1.2465), was used as a model organism in inhibitory tests. Bacteria were grown at 37 °C in Luria-Bertani (LB) medium to logarithmic phase (absorbance of 0.15 at 600 nm on SPECTROstar Omega plate reader, BMG, Germany), about 10^8^/mL bacteria. Afterwards, 1 ml of the bacterial suspension was centrifuged at 5000 r/min for 10 min, and resuspended in 1 ml distilled water. Then, 100 μl of the resulting *S. aureus* suspension was added into 10 ml PAW equilibrated at 37 °C (5 min), for 20 min incubation. *S. aureus* suspension treated with PAW-0 was setup as the negative control. All experiments were repeated three times for statistical analysis.

### Sterilization Ability of PAW

Colony count assay was used to assess kinetic killing curves and antimicrobial effects of a given compound or device. In brief, after 20 min PAW treatment, tenfold serial dilutions of 100 μl treated suspension were plated on LB agar culture medium, and incubated at 37 °C for about 21 h before CFU count. The sterilization ability of PAW was evaluated by the Log Reduction, calculated as follows:


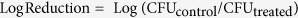


Scanning electron microscope (SEM, Quanta 200FEG) was used to evaluate cell morphology before and after PAW treatment. For SEM sample preparation, cells were pelleted by centrifugation (5000 rpm), and dehydrated by alcohol gradient (30, 50, 70, 80, 90, and 100%) until desiccation. The samples were then gilded and observed under an electron microscope.

### OES Detection of the Main Excited ROS in PAW

To identify the main excited reactive species in plasma activated water, OES was employed in the 200–1000 nm range along the axial direction of the PMJ with the AvaSpec-2048-8 Fiber Optic. Plasma discharge formed beneath the water surface and one end of the fiber optics cable was used to acquire light signals at the bottom of the water container (quartz tube), approximately 5 mm away from the exit nozzle^30^.

### Evaluation of PAW’s Physical and Chemical Properties

After PAW storage at different temperatures for various times, pH, ORP, and H_2_O_2_ contents were immediately detected to evaluate its physical and chemical properties. ORP, an indicator of the ability of a solution to oxidize and related to oxidizer concentration, activity, or strength^31^, was employed to evaluate global reactive oxygen species (ROS) levels in PAW. ORP in each sample was measured with a redox sensitive electrode (Mettler-Toledo LE501). H_2_O_2_ concentrations in PAW samples were quantified by Hydrogen Peroxide Assay Kit (Beyotime, Jiangsu, China); pH was monitored by a microprocessor pH-meter (Mettler-Toledo LE438).

### Detection of nitrate and nitrite in PAW

The concentrations of nitrate anions (NO_3_^−^) and nitrite anions (NO_2_^−^) in PAW samples were measured by spectrophotometry^32^,^33^,^34^. After PAW storage at different temperature, 10 ml PAW were added 200 μl of 1 mol/L hydrochloric acid and 20 μl of 0.8% sulfamic acid. NO_3_^−^ levels in PAW samples were determined by ultraviolet absorption spectrometry (NanoDrop 8000) at a single wavelength of 220 nm. As for NO_2_^−^, sulphanilamide was used as the diazotizing reagent, and N-(1-naphthy1)-ethylenediamine hydrochloride as the coupling reagent. After plasma activation, 200 μl of 10 g/L sulphanilamide was added into 10 ml PAW, and incubated at room temperature for 2 min, subsequently adding 200 μl of 1.0 g/L N-(1-naphthy1)-ethylenediamine hydrochloride for 20 min at room temperature. After incubation, NO_2_^−^ levels were measured at 540 nm.

### Measurement of NO radicals

N-methyl-D-glucamine dithiocarbamate (MGD) (99%, J&K Scientific Ltd. China) and FeSO_4_·7H_2_O (AR, National Medicine Group Chemical Reagent Co. Ltd) were used to trap NO free radicals produced in PAW by ESR spectroscopy, which result in the stable paramagnetic complex NO-Fe^2+^(MGD)_2_ with a characteristic ESR feature. After PAW melting, 250 μl MGD (1 mol/L) and 250 μl Fe^2+^ (0.3 mol/L) were added into 0.5 ml samples immediately. The reaction was initiated by addition of iron. The final product was imbibed by a capillary and detected in the resonator cavity of an ESR spectrometer (ER-200D-SRC/E-500, Bruker Ltd, German) operated at room temperature. The nitric oxide donor sodium nitroprusside (SNP, Beyotime, Jiangsu, China) was detected by ESR spectroscopy as standard for assessing NO radical concentrations in PAW. ESR experiments were carried out under following conditions: central magnetic field, 3369.850 Gauss; sweep width, 100.0 Gauss; frequency, 9.628 GHz; modulation frequency, 100 kHz; power, 7.971 mW.

### Statistical Analysis

Data were obtained from at least three independent experiments (n ≥ 3). Values are mean ± standard deviation (SD). Statistical analysis was performed using SPSS statistical package 17.0 (SPSS Inc., USA). Analysis of variance (ANOVA) was used to compare different treatments during PAW storage; significant differences were identified by the Student-Newman-Keuls multiple range test, with a confidence level at P ≤ 0.05. In addition, paired-samples t-test was employed to compare the effects of different treatment conditions on NO radicals in PAW after 30 days of storage. Significant differences were represented by *P < 0.05, **P < 0.01, and ***P < 0.001.

## Results and Discussion

### Bactericidal efficiency of PAW samples under different storage temperatures

#### S. aureus inhibition

**Figure 2:**
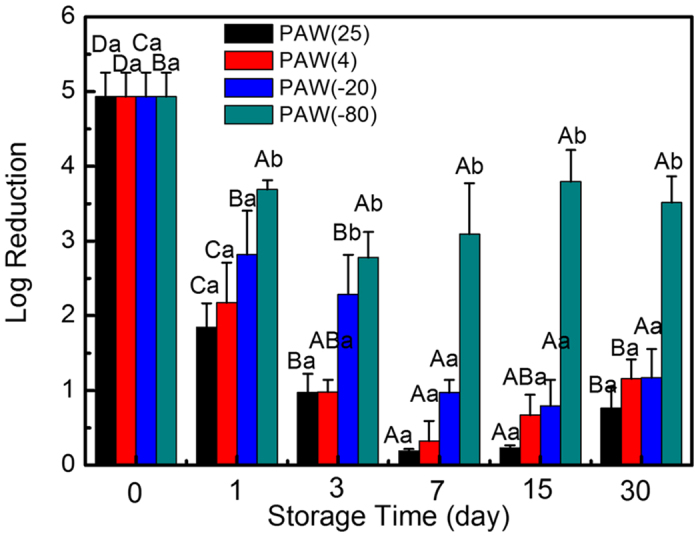
Inactivation rate of *S. aureus* treated with PAW stored at different temperatures for 30 days. Different lowercase letters for the same storage time indicate significant differences (p < 0.05). Different capital letters for the same storage temperature but different times indicate significant differences (p < 0.05).

The colony count assay was employed to assess the bactericidal effects of PAW samples stored at different temperatures for various days. Bactericidal ability of PAW increased with decreasing temperature, with −80 > −20 > 4 > 25 (°C) obtained in descending order for bacterial inhibition. Before storage, fresh PAW resulted in a log reduction of approximately 5 for *S. aureus*. As shown in Fig. 2, bacterial growth declined to 3.7 and 1.8 at −80 °C and 25 °C, respectively, after 1 day of storage. PAW stored at −80 °C retained higher antibacterial activity compared to the other temperatures, leading to a log reduction of 3~4 for *S. aureus*; at other temperatures, PAW reduced total bacterial growth by 1.8, 2.2, 2.9 logs compared with untreated control, which decreased to about 1 log after 30 days of storage. Generally speaking, significant differences in bacterial killing were obtained between PAW(−80) and PAWs stored at other temperatures. These findings indicated that loss of bactericidal activity in PAW(−80) was reduced compared with that of other temperatures, indicating PAW(−80) retained efficient content, and consequently bactericidal activity. The differences for various storage temperatures might be explained by great variations in the physicochemical properties of PAW in these conditions. These results were consistent with a previous research assessing acidic electrolyzed water (AEW), and revealing that storage at −18 °C may be a better method to retain the available chlorine concentration as well as bactericidal activity compared to storage at 25 °C^35^.

#### SEM assessment of S. aureus

**Figure 3:**
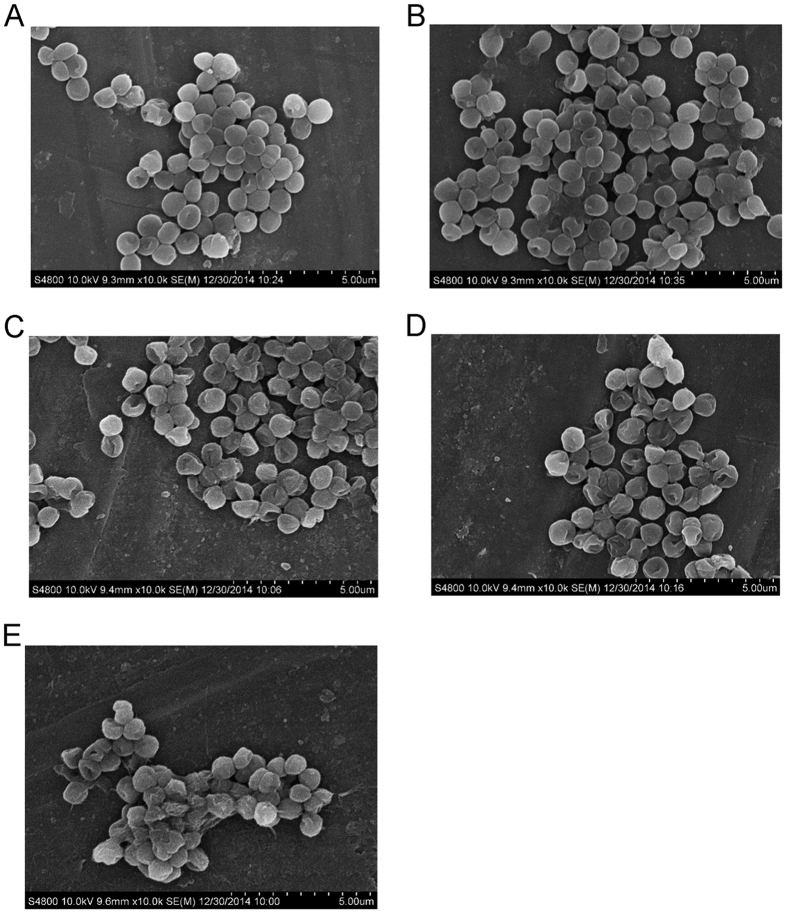
Scanning electron microscope (SEM) images of *S. aureus* after PAW treatment for 20 min. (**A**) Untreated; (**B**) 25 °C; (**C**) 4 °C; (**D**) −20 °C; (**E**) −80 °C for 30 days, respectively (magnification x10000).

Scanning electron microscope (SEM) study further verified the stronger bactericidal ability of PAW(−80) compared to that of other temperatures. Typical SEM images of *S. aureus* before and after 20 min of PAW treatment following 30-day storage are shown in Fig. 3. Interestingly, the bacteria underwent a transition from initially smooth surfaces to surfaces with distortion, shrinkage and rupture of the outer layer after PAW treatment. The level of damage depended on storage temperature, and was more severe for −80 °C (Fig. 3(E)) compared to 25 °C (Fig. 3(B)), 4 °C (Fig. 3(C)), and −20 °C (Fig. 3(D)). The bacteria underwent a transition from smooth (Fig. 3(A)) to severely deformed (Fig. 3(E)) surfaces. These results were consistent with bacterial number reduction (Fig. 2), indicating the superiority of PAW stored at −80 °C over samples kept at other temperatures.

### Physicochemical properties of PAW during storage

#### OES Detection of Main Excited ROS in PAW

**Figure 4:**
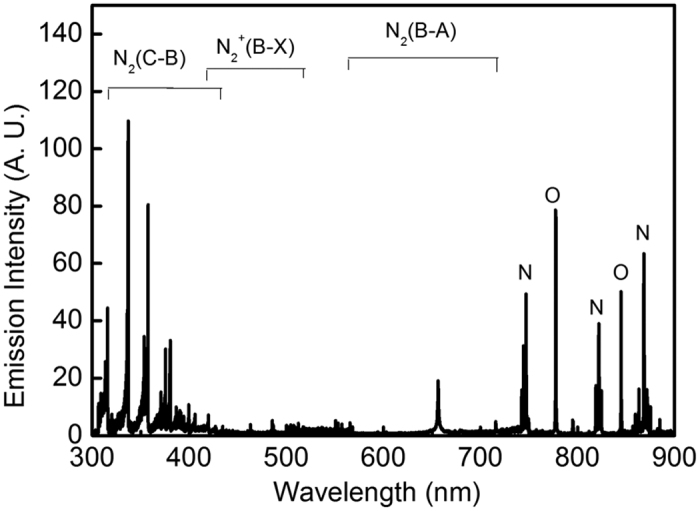
End-on optical emission spectra of PMJ ranging from 300 to 900 nm operated in water.

OES was employed to investigate the main excited active species generated in PAW. In the end-on spectra of the PAW, PMJ operated beneath the water surface at an operating current of 35 mA and voltage of 100 V (Fig. 4(A)). OES results are dominated by the second positive system of 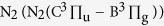, first negative system of 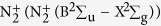, and first positive system of 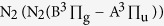 at 300–420 nm, 400–520 nm, and 560–730 nm, respectively, as well as N with O emission lines at 730–900 nm. O and N generated from discharge are mainly formed through dissociation of O_2_ and N_2_, respectively, which are excited from the ground state by electron impact (1) and (2)^29^.


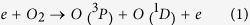



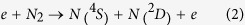


OES data indicated that excited atomic oxygen and nitrogen were produced in water by the plasma. Atomic oxygen is a chemically reactive species which can cause damage in biological molecules^36^. Atomic oxygen is readily converted into other reactive oxygen species, such as O_3_, ·OH and H_2_O_2_, due to high activity through chemical reactions^37^,^38^,^39^. Nitrites, nitrates, S-nitrosothiols and nitrosamines are metabolites of NO and mediators of the related cytotoxic effects, namely inhibition of mitochondrial respiration DNA damage leading to gene mutation, protein alteration and loss of function, necrosis, and apoptosis^40^.

#### PH and ORP in PAW samples

**Figure 5:**
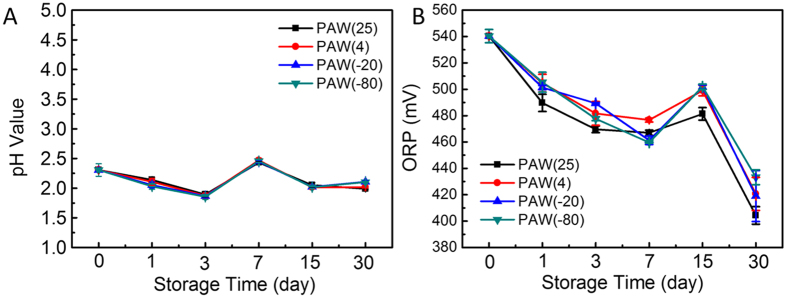
Changes of (**A**) pH and (**B**) ORP of PAW stored at different temperatures over storage time.

Physicochemical parameters of PAW showed significant differences at all storage temperatures compared with the control group, PAW-0. Solution pH and ORP were obtained during the storage process (Fig. 5). After plasma activation for 20 min, PAW’s pH decreased to 2.3 from 6.8, while ORP reached about 540 mV from the initial 250 mV. On the other hand, there was no significant change among the four different storage temperatures for the same time. As shown in Fig. 5, pH values of all samples only slightly changed with increasing storage time, and remained essentially stable during the 30-day storage in all four conditions; the pH of PAW remained around 2.0 regardless of storage temperature.

ORP is regarded as an important factor influencing microbial inhibition. A high ORP can damage the outer and inner membranes^41^. Thus, it is of great significance to explore changes in PAW ORP for monitoring bactericidal efficacy. Figure 5(B) shows ORP for PAW samples stored under four different conditions for 30 days, respectively. As for different temperatures, changes followed a similar trend; they decreased slightly during the initial seven storage days by 15.6%, 13.3%, 17.1%, and 17.5%, respectively, and were reduced by 15.6%, 13.3%, 10.0%, and 6.2%, respectively, from days 7 to 30.

The synergetic effects of acidic pH and ROS always provide a warranty of relatively good germicidal efficacy^6^. In addition, high ORP indicates a solution with high oxidative strength^14^. There was no obvious change between different temperatures, demonstrating that they are not pivotal factors in sterilizing against *S. aureus* among different storage conditions.

#### H_2_O_2_ Concentrations in PAW samples

**Figure 6:**
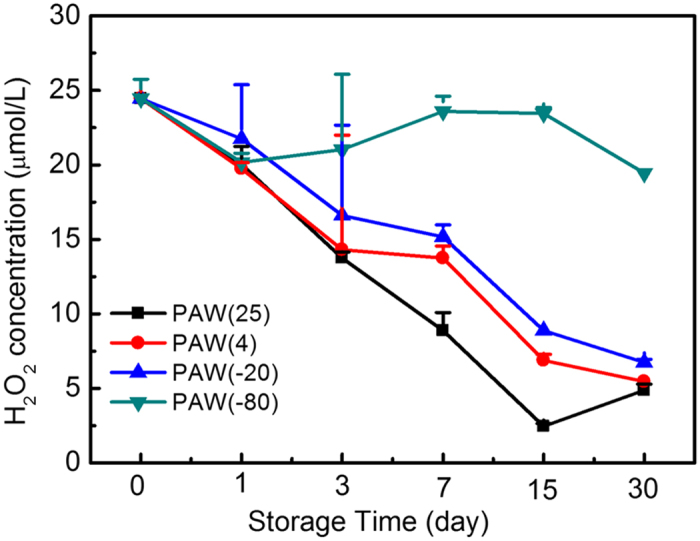
H_2_O_2_ concentration in PAW samples stored at different temperatures and times.

H_2_O_2_ is thought to be involved in the antimicrobial properties of PAW, and previous investigations have assessed the antimicrobial contributions of H_2_O_2_ in PAW. R. Burlica^42^ observed that approximately 3 mM H_2_O_2_ contributes a 2-log reduction in bacterial colony formation, while M. Naitaliet^6^ noted that 10 μM acidified H_2_O_2_ yields a 0.4-log reduction. We measured the H_2_O_2_ concentration of PAW immediately, approximately 24.4 μM (Fig. 6). H_2_O_2_ concentrations in PAW stored at −80 °C were approximately 20 μM and did not vary significantly over 30 days. With regard to PAW samples stored at other temperatures, H_2_O_2_ levels decreased to 6.0 μM after 30 days of storage from approximately 24.4 μM, indicating that H_2_O_2_ in PAW has reacted with various substances under such conditions. H_2_O_2_ is considered a strong oxidizer, especially in acidic environments^43^; thus loss of H_2_O_2_ might play an important role in different disinfection efficiencies observed.

#### Nitrate and Nitrite Concentrations in PAW

**Figure 7:**
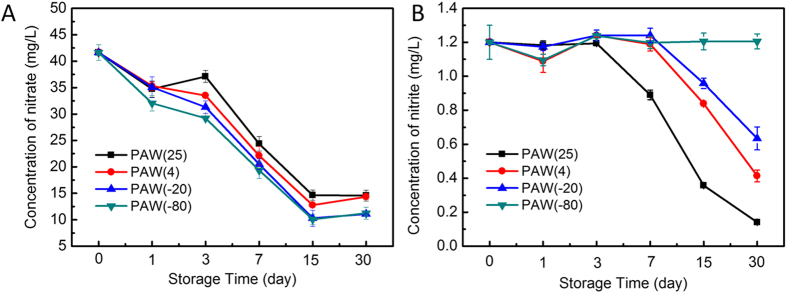
Concentration of (**A**) nitrate and (**B**) nitrite in PAW stored at different temperatures and times.

In addition to ROS, reactive nitrogen species (RNS) such as nitrites, nitrates and peroxynitrites have also attracted attention from scientists for their sterilization mechanisms in PAW^20^,^44^,^45^,^46^,^47^. Formation of nitrogen transient species was evidenced mainly indirectly through detection of nitrogen products NO_2_^−^ and NO_3_^−^ in plasma treated liquid. Thus, NO_3_^−^ and NO_2_^−^ levels in PAW were also monitored by ultraviolet spectrophotometry. As shown in Fig. 7(A), NO_3_^−^ concentrations had a similar trend in PAW samples for all conditions, decreasing with storage time. As for storage temperature, no significant differences in NO_3_^−^ concentrations among various storage conditions were obtained. With respect to NO_2_^−^, concentrations in PAW(−80) were effectively constant over 30 days, about 1.2 μM. However, nitrite levels in PAW varied, decreasing from 1.2 μM to 0.1 μM, 1.2 μM to 0.4 μM, and 1.2 μM to 0.6 μM at 25 °C, 4 °C, and −20 °C, respectively, within 30 days. Meanwhile, nitrite levels were highly related to storage temperature, decreasing along with increasing temperature. Furthermore, significant differences in NO_2_^−^ concentrations between PAW(25) and PAW(−80) were obtained (p < 0.05). In addition, the post-discharge reaction between hydrogen peroxide and nitrite ions occurring in water after treatment with plasma determined the formation of peroxynitrite: NO_2_^−^ +H_2_O_2_+H^+^→ONOOH + H_2_O^48^. Peroxynitrite chemistry was shown to significantly participate in the antibacterial properties of PAW^6^,^8^,^18^,^19^. NO_3_^−^ amounts in PAW samples significantly decreased after storage, while NO_2_^−^ and H_2_O_2_ amounts dropped faster after storage at the other three temperatures compared with −80 °C, indicating that PAW(−80) is more favorable to keep NO_2_^−^, H_2_O_2_, and consequently, bactericidal activity. For solutions treated by liquid-phase plasmas, the antimicrobial properties of PAW were tentatively attributed to the synergetic effects of H_2_O_2_ and nitrite remaining in noticeable concentrations. This would be good news for users in practical applications, because PAW(−80) can avoid active ingredient loss during storage.

#### NO radical amounts in PAW samples

**Figure 8:**
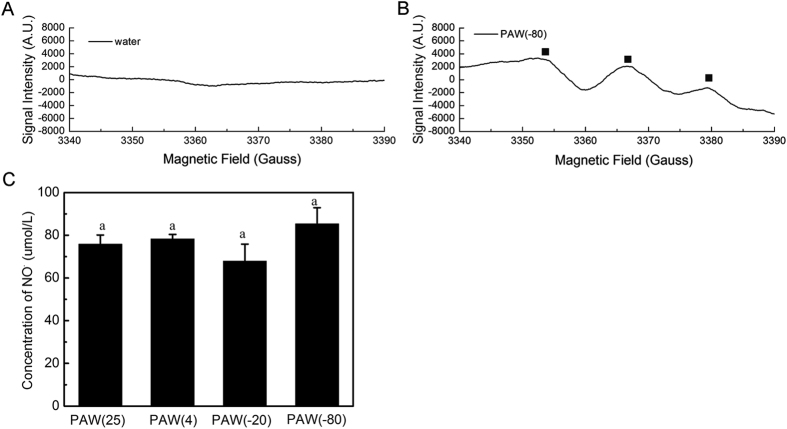
Electron spin resonance (ESR) spectrum of MGD-NO signal in PAW(−80) and NO concentrations in PAW stored at different temperature for 30 days. Same lowercase letters for the 30^th^ indicate insignificant differences (p > 0.05).

The direct spin trapping reaction between Fe^2+^(MGD)_2_ and NO produces the spin adduct NO-Fe^2+^(MGD)_2_ that is characterized by a tripling ESR spectrum, with a peak intensity ratio of 1:1:1. As shown in Fig. 8(A,B), NO was detected in PAW but virtually absent in the PAW-0 control. NO-Fe^2+^(MGD)_2_ signals detected in PAW demonstrated that NO radical is generated in solutions activated by plasma. After 30-day storage, relative NO-Fe^2+^(MGD)_2_ signal intensities for the same experimental conditions in PAW(25), PAW(4), PAW(−20) and PAW(−80) achieved 75.84, 78.34, 67.91, 85.28 μmol/L, respectively, according to the standard concentration of NO donor. There were no significant differences among treatment groups, indicating that NO radical is not a key factor affecting sterilization ability of PAWs stored at different temperatures. However, potential antimicrobial effects of NO have also been reported. T. Lai^49^ observed that exogenous NO can induce generation of ROS and cause oxidative damage to proteins during the germination process, resulting in growth inhibition of *Penicillium expansum*. After PAW storage, reactive nitrogen oxide species (RNOS) cause oxidative and nitrosative damage by altering DNA, inhibiting enzyme function, and inducing lipid peroxidation, which account for the majority of NO’s antimicrobial properties. The results demonstrated that NO radicals play a pivotal role in *S. aureus* inhibition, nearly independently of temperature.

### Pearson correlation analysis of ORP, H_2_O_2_, and NO_3_^−^, and *S. aureus* inhibition along with storage time

The results demonstrated that bactericidal ability of PAW(−80) remained stable during storage, while that of PAW(−20), PAW(4), PAW(25) displayed a decreasing trend with increasing storage time. The Pearson’s correlations between physicochemical parameters and *S. aureus* growth reduction induced by PAW(25), PAW(4), PAW(−20) during 30 days of storage were evaluated, respectively. As shown in Table 1, Pearson correlation coefficients in PAW(25), PAW(4), and PAW(−20) in association with ORP were 0.729, 0.661, and 0.723, respectively; those between the above treatment groups and H_2_O_2_ were 0.851, 0.783, and 0.861, respectively. With regard to NO_3_^−^, Pearson correlation coefficients were 0.727, 0.742, and 0.905, respectively. Significantly positive correlation was obtained during the whole storage period relative to pH and NO_2_^−^. In general, PAW’s inhibitory activity changed with storage time, and depended on three germicidal factors, specifically ORP, H_2_O_2_, and NO_3_^−^. ORP is considered an important factor affecting microbial killing as it can damage outer and inner membranes of *E. coli* O157:H7, subsequently leading to inhibition^41^. H_2_O_2_ is considered an important constituent in sterilization. Nitrate, as a long-lived secondary product, is likely responsible for the extended biological effects of plasma-activated water after plasma treatment^8^. ORP, H_2_O_2_ and their combination with nitrates have been proposed to be the dominant factors in PAW(25), PAW(4), and PAW(−20) affecting microbial killing over storage time.

### Comparisons of physicochemical properties of PAW stored at different temperatures for 30 days

To the best of our knowledge, whether storage temperature significantly affects bactericidal activity of PAW remains unclear. In this study, sterilization efficiency of PAW(−80) was better compared to values obtained for other treatment groups, with PAW(−80) > PAW(−20) > PAW(4) > PAW(25). These differences might be explained by distinct physicochemical properties of PAW after storage at various temperatures.

As shown in Table 2, ORP in different PAWs remained almost unchanged after 30 days of storage, with no significant differences among storage temperatures (p > 0.05). However, pH and nitrate of PAW(−80) and PAW(−20) significantly differed from values obtained for PAW(4) and PAW(25) (<0.05); the differences between PAW(−80) and PAW(−20) were insignificant (p > 0.05). Interestingly, PAW(−80) showed significant differences compared with PAW samples stored at other temperatures (25, 4 or −20 °C), specifically H_2_O_2_ and NO_2_^−^. Both H_2_O_2_ and NO_2_^−^ levels in PAW(−80) remained stable, while the other treatment groups showed decreased levels as shown in Table 2. H_2_O_2_ amounts in PAW(−80) were 3.2, 2.9, an 1.8 times, respectively, higher compared with PAW(25), PAW(4) and PAW(−20) values at the end of storage. Similarly, nitrite ion concentrations in PAW(−80) were significantly different from those of other treatment groups (<0.05). It is possible that the reaction between acidified nitrites and 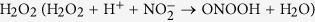 is affected by temperature. Nitrite production may thus be transiently encountered and biologically active in PAW.

Our results indicated that bactericidal activity could be preserved at −80 °C during PAW storage. Consequently, −80 °C has the potential to keep freshness and product sanitization in melted PAW. Compared with PAW samples stored at other temperatures, physicochemical parameters of PAW(−80) had significant changes (Table 2). This is good news for PAW users, since in practice PAW can be prepared and stored at −80 °C until needed, avoiding loss of constituents during storage. Furthermore, melted PAW can be treated as common water because of reduced adverse impact on the human body as well as the environment.

## Conclusion

We demonstrated that PAW(−80) is more efficient than PAWs stored at other temperatures (25 °C, 4 °C, and −20 °C) for sterilization. With regard to pH, ORP, and NO_3_^−^, minimal changes of PAW were observed. H_2_O_2_ and nitrite concentrations presented the same trend, decreasing with increasing storage temperature. Evaluation of NO radicals by ESR showed that short-lived species slightly contribute to the differences in sterilization efficiency of PAW stored at different temperatures. This suggested that PAW(−80) can preserve bactericidal activity, and H_2_O_2_ and NO_2_^−^ levels, leading to *S. aureus* inhibition.

This work unravels the importance of storage temperature on physicochemical properties and bactericidal efficiency of PAW, providing a basis for practical application of PAW in inactivating harmful microorganisms in medicine and foodstuff. In the future, other storage conditions should be assessed, e.g. light, agitation, and packaging.

### Statement of Novelty

Plasma-activated water (PAW) as a novel disinfectant can be used to inhibit harmful microorganisms in food industry and medical settings. However, the short life of active species in PAW is an important limitation for practical applications. Here, physicochemical properties and bactericidal activities of PAW stored at 25, 4, −20, −80 °C, respectively, were assessed. Results showed PAW stored at −80 °C retained efficient inhibitory activity against *S. aureus*. Moreover, NO_2_^−^ contributed to the antimicrobial property, in combination with H_2_O_2_. These findings provide a basis for PAW storage and practical applications in food and medical industry.

### Highlights

PAW stored at −80 °C retains bactericidal activity.

NO_2_^−^ contributes to the antibacterial property of PAW(−80) in combination with H_2_O_2_.

Bactericidal ability of PAW stored at 25, 4, and −20 °C decreased over time.

Disinfection properties depended on ORP, H_2_O_2_, and NO_3_^−^.

## Additional Information

**How to cite this article**: Shen, J. *et al*. Bactericidal Effects against *S. aureus* and Physicochemical Properties of Plasma Activated Water stored at different temperatures. *Sci. Rep.* **6**, 28505; doi: 10.1038/srep28505 (2016).

## Figures and Tables

**Table 1:** Pearson correlation coefficients between ORP, H_2_O_2_, and NO_3_^− +^, and inactivation of *S. aureus* for PAW(25), PAW(4), and PAW(−20) treatment during 30 days of storage.

Treatments	Pearson correlation coefficient				
	pH	ORP	H_2_O_2_	NO_3_^−^	NO_2_^−^
PAW(25)	0.307	0.729	0.851	0.727	0.530
PAW(4)	0.259	0.661	0.783	0.742	0.274
PAW(−20)	0.134	0.723	0.861	0.905	0.439

**Table 2:** Physicochemical properties, including ORP, H_2_O_2_, and NO_3_^−^ for PAW(25), PAW(4), PAW(−20) and PAW(−80) for 30 days.

Treatments	Physicochemical properties				
	pH	ORP	H_2_O_2_	NO_3_^−^	NO_2_^−^
PAW(25)	1.99 ± 0.01a	404 ± 7a	4.60 ± 0.57a	14.57 ± 1.03b	0.14 ± 0.01a
PAW(4)	2.02 ± 0.02a	421 ± 13a	4.98 ± 0.44a	14.35 ± 0.55b	0.41 ± 0.03b
PAW(−20)	2.11 ± 0.02b	419 ± 19a	6.98 ± 0.44b	11.09 ± 0.13a	0.63 ± 0.07c
PAW(−80)	2.10 ± 0.02b	433 ± 6a	19.46 ± 0.29c	11.24 ± 1.13a	1.21 ± 0.04d

Different lowercase letters indicate significant differences (p < 0.05).
